# Divergent paths: *CAP59* gene evolution in Cryptococcus and implications for pathogenicity

**DOI:** 10.22034/cmm.2024.345180.1496

**Published:** 2024-04-15

**Authors:** Noor Maath Ahmed, Ahmed AbdulJabbar Suleiman

**Affiliations:** 1 Department of Biology, College of Science, Tikrit University, Tikrit, Saladin, Iraq; 2 Department of Biotechnology, College of Science, University of Anbar, Ramadi, Anbar, Iraq

**Keywords:** *CAP59*, *Cryptococcus* virulence, Virulent *Cryptococcus*, Non-virulent *Cryptococcus*, Pathogenicity, Bioinformatics analysis

## Abstract

**Background and Purpose:**

*Cryptococcus neoformans* and *Cryptococcus gattii* are highly virulent species that cause diseases, such as meningoencephalitis and pulmonary infections. The *CAP59* gene predominantly determines the virulence of the pathogenic species. This study aimed to examine *CAP59* in both pathogenic and non-pathogenic species.

**Materials and Methods:**

This study identified *Cryptococcus* species through extensive literature, retrieved sequences from UniProt, explored protein families utilizing InterPro, motif analysis by MEME, multiple sequence alignment using Clustal Omega, performance of the phylogenetic analysis with MEGA, modeled protein structures with MODELLER, and separately visualized pathogenic and non-pathogenic structures in PyMOL.

**Results:**

Motif analysis showed four conserved regions between the pathogenic and non-pathogenic sequences. Moreover, multiple sequence alignment revealed that pathogenic *CAP59* gene sequences lacked a significant portion, compared to non-pathogenic ones, with several mutations in the gene sequence of pathogenic species *CAP59* at highly conserved regions. The phylogenetic analysis and pairwise distance matrix revealed that *Cryptococcus amylolentus* is closely related to pathogenic species. Predicted *CAP59* protein structures were superimposed to show structural differences between pathogenic and non-pathogenic species.

**Conclusion:**

In conclusion, the results suggested that non-pathogenic species may have evolved into pathogenic species since the *CAP59* gene sequences of the non-virulent species were longer than those of the virulent species sequences. It implies that the virulent sequences may have lost that region at some point in evolution, which additional research on capsule formation-related genes can further corroborate.

## Introduction

*Cryptococcus* is a fungus of the phylum Basidiomycota, order Sporidiales, and family Sporidiobolaceae, which comprises more than 100 species widespread in the environment [ 1 ]. The environmental sources for these species are air, soil, water, bird excreta, animals, and decomposing wood [ 2 ]. Furthermore, only a few species within the *Cryptococcus* genus show medical significance, causing virulence [ 3 ]. Among these *Cryptococcus* species, the two predominant species responsible for diseases in animals and humans are *Cryptococcus neoformans* and *Cryptococcus gattii* [ 4 ]. *Cryptococcus neoformans* infections are common in immunocompromised individuals, causing meningoencephalitis. However, *C. gattii* more often affects immunocompetent individuals, leading to pulmonary infections [ 5 , 6 ]. Additionally, these infections can disseminate widely, causing skin, eye, and prostate infections in both immunocompromised and immunocompetent patients [ 7 ].

Timely and accurate diagnosis of cryptococcal disease is crucial for patient outcomes. The main diagnostic methods include microscopy for *Cryptococcus* identification and capsular antigen detection. Clinical specimens commonly used for *Cryptococcus* detection are from the neurological, hematological, or respiratory systems [ 8 ]. However, the infection site, immune status of the patients, and severity of the symptoms caused by cryptococcal infections suggest the treatment of the infections [ 9 ]. Non-immunosuppressed patients with suspected pulmonary cryptococcal infection can receive 400 mg of fluconazole daily for 6-12 months. This treatment is also advised for non-meningeal and non-pulmonary cryptococcosis after ruling out the central nervous system disease. While those who are immunosuppressed need a lumbar puncture to treat asymptomatic infections [ 10 ].

The infections caused by *C. neoformans* and *C. gattii* can be fatal if the patient does not receive the appropriate treatment [ 11 ]. Cryptococcal meningitis causes about 181,000 deaths every year; 80 % of cases are due to *C. neoformans*, and infections with *C. gattii* are more common in tropical and subtropical areas [ 12 ].

The capsule of *C. neoformans* is a crucial component of its pathogenicity, mainly consisting of glucuronoxylomannan and galactoxylomannan [ 13 , 14 ]. This species has four serotypes, namely A, B, C, and D. Serotypes B and C are called *C. neoformans* var. *gattii*, and serotypes A and D are *C. neoformans* var. *neoformans*. It is accepted that *C. gattii* is distinct from *C. neoformans* var. *gattii* [ 15 ]. The *C. gattii* species complex comprises four major molecular types, namely VGI, VGII, VGIII, and VGIV [ 16 ]. Furthermore, various non-pathogenic *Cryptococcus* species, such as *C. amylolentus*, *C. wingfieldii*, *C. depauperatus*, *C. floricola*, *C. luteus*, *C. bestiolae*, and *C. dejecticola* are identified. Among these non-pathogenic species, it is reported that the pathogenic species have evolved from *C. amylolentus* species [ 17 - 23 ].

A capsular formation-associated gene, *CAP59*, and its function is reported in *Cryptococcus* species; however, its role in *C. gattii* is less studied, while the capsule interferes with the phagocytosis and clearance of macrophages in the immune system by providing a physical barrier [ 24 ]. Expansion of the capsule and alterations in its structure, density, and size in the host optimize the survival opportunities of *Cryptococcus* species [ 25 , 26 ].

Hence, this study aimed to investigate the evolutionary relation of the *CAP59* gene in *Cryptococcus* species. Computational analyses, including motif identification, multiple sequence alignment (MSA), phylogenetic analysis, pairwise distance matrix, and superimposition of *CAP59* gene structures, aimed to reveal molecular divergence mechanisms and insights into the virulence of pathogenic *Cryptococcus* species.

## Materials and Methods

### Cryptococcus Species Identification

An extensive literature review retrieved pathogenic and non-pathogenic *Cryptococcus* species using a systematic search across databases with terms, like "*Cryptococcus* species," "pathogenic *Cryptococcus*," and "non-pathogenic *Cryptococcus*." 

### Cryptococcus Species Sequences Retrieval

Amino acid sequences of pathogenic *Cryptococcus* species were obtained from UniProt. However, for non-pathogenic sequences, the Basic Local Alignment Search Tool (BLAST), which finds local similarities between protein or nucleotide sequences [ 27 ], was utilized to identify sequences similar to the pathogenic species and retrieve non-pathogenic species sequences.

### Proteins Family Analysis

Protein family analysis for pathogenic and non-pathogenic *Cryptococcus* species was conducted using Pfam, a widely used database categorizing protein sequences into families and domains [ 28 , 29 ]. Pfam was accessed through the InterPro database, and the UniProt IDs of the *CAP59* gene of pathogenic and non-pathogenic species were searched on the InterPro database to describe the protein families.

### Motif Analysis

Motif analysis of the retrieved sequences was performed using Multiple Expectation maximization for Motif Elicitation (MEME), a widely used tool for searching for novel ‘signals’ in sets of biological sequences [ 30 ]. All the FASTA sequences of the pathogenic and non-pathogenic species were collected in a single file for input in the MEME web server.

### Sequence Alignment of Cryptococcus Species

The MSA was performed using Clustal Omega, which is used to align protein sequences and deliver accurate alignments [ 31 ]. Sequences of pathogenic species were aligned to identify conserved, semi-conserved, non-conserved, or identical regions. Nonpathogenic sequences were aligned separately. Finally, both pathogenic and non-pathogenic sequences were aligned together.

### Phylogenetic Analysis

The phylogenetic analysis of the aligned sequences was performed using Molecular Evolutionary Genetics Analysis (MEGA) software https://www.megasoftware.net/, which contains a large collection of methods and tools for computational molecular evolution [ 32 ]. The aligned sequences were entered into the MEGA software to construct a maximum likelihood phylogenetic tree; additionally, pairwise distance analysis was performed, generating a matrix.

### Gene Structures Retrieval

The *CAP59* gene structures for all the *Cryptococcus* species (pathogenic and non-pathogenic) were modeled through homology modeling by retrieving a single template for pathogenic and non-pathogenic species, respectively. The AlphaFold database [ 33 ] was utilized to retrieve structures of *C. neoformans* for pathogenic species and *C. wingfieldii* for non-pathogenic species. Furthermore, MODELLER 10.3, a program for homology modeling [ 8 ], was used to model the structures using a Python script for MODELLER.

### Visualization and Superimposition of Structures

The modeled structures were visualized using PyMOL (PyMOL Molecular Graphics System, Version 2.0 Schrödinger, LLC), a cross-platform graphics tool widely used for three-dimensional visualizations of proteins, nucleic acids, small molecules, electron densities, surfaces, and trajectories [ 34 ]. The pathogenic and non-pathogenic structures were superimposed separately to visualize the non-conserved regions between pathogenic and non-pathogenic structures, respectively.

## Results

### Cryptococcus Species Identification

In total, 39 *Cryptococcus* species were identified through an extensive literature review, while among the 39 species, 12 were pathogenic, and 27 were non-pathogenic *Cryptococcus* species. The pathogenic and non-pathogenic species are mentioned in supplementary document Table S1.

### Cryptococcus Species Sequences Retrieval

The UniProt database contained *CAP59* gene sequences for both pathogenic and non-pathogenic *Cryptococcus* species. Only 7 pathogenic species out of 12 had available sequences, while none of the 27 non-pathogenic species was available on the UniProt database. BLAST searches using pathogenic species yielded sequences for four non-pathogenic species, namely *Cryptococcus wingfieldii* CBS 7118, *Cryptococcus depauperatus* CBS 7855, *Cryptococcus floricola*, and *Cryptococcus amylolentus* CBS 6039. The retrieved sequences and the UniProt IDs of the pathogenic and non-pathogenic species are mentioned in supplementary document Table S2.

### Protein Family Analysis

The protein family analysis of the pathogenic and non-pathogenic species was performed using the Pfam database, revealing the *CAP59* gene family regions. The pathogenic species, *C. neoformans*, *C. gattii* VGI, and *C. gattii* VGIV/VGIIIc, showed two protein family regions, namely receptor family ligand-binding region and cryptococcal mannosyltransferase 1. However, the pathogenic species, *C. gattii*, *C. gattii* VGII, *C. gattii* VGIIb, and *C. gattii* VGIV showed only a single region, cryptococcal mannosyltransferase 1. Furthermore, the non-pathogenic species also showed cryptococcal mannosyltransferase 1 region at different positions. Details of the protein family analysis of pathogenic and non-pathogenic species are mentioned in supplementary document Table S3.

### Motif Analysis

**Figure 1:**
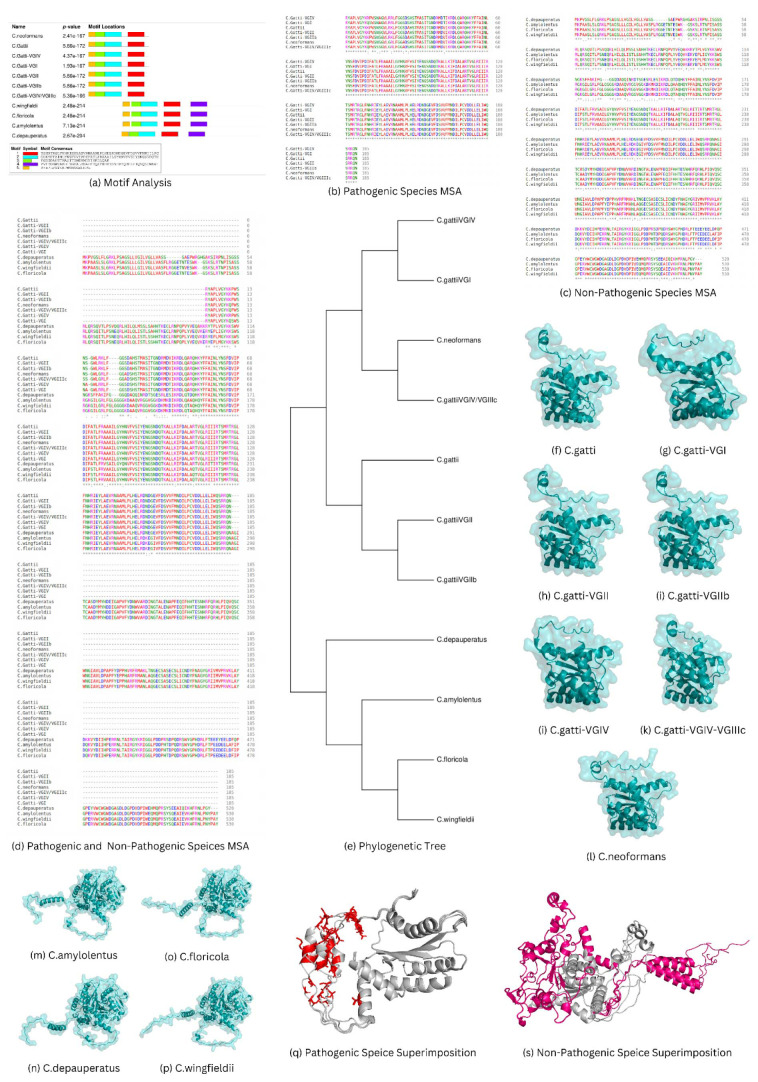
(a) Motif analysis of the pathogenic and non-pathogenic *Cryptococcus* species, (b) Pathogenic *Cryptococcus* species multiple sequence analysis (c) Non-pathogenic *Cryptococcus* species multiple sequence analysis, (d) multiple sequence analysis of the *CAP59* gene of the pathogenic and non-pathogenic *Cryptococcus* species, (e) Phylogenetic analysis of the pathogenic and non-pathogenic species performed using MEGA software, (f-p) An overview of the pathogenic and non-pathogenic structures of *Cryptococcus* species visualized in PyMOL software, (q-r) Superimposition of the pathogenic and non-pathogenic species structures visualized in PyMOL. The mutations colored are red and pink.

The MEME tool analyzed *CAP59* gene sequences, identifying four pathogenic and five non-pathogenic motifs. Among pathogenic sequences, motifs at positions 1-21, 22-50, 51-100, and 121-170 remained consistent, whereas non-pathogenic motifs varied. *Cryptococcus wingfieldii*, *C. floricola*, *C. amylolentus* and *C. depauperatus* had motifs at positions 106-126, 132-160, 161-210, and 231-280, respectively, with an additional motif at 313-362. In contrast, *C. depauperatus* displayed motifs at positions 102-122, 125-153, 154-203, 224-273, and 306-355. The motif regions of the *CAP59* gene for the pathogenic and non-pathogenic species are shown in Figure 1a.

### Sequence Alignment of Cryptococcus Species

The MSA of the pathogenic sequences, using Clustal Omega, revealed that most of the sequences were conserved except for the residues at positions 10, 11, 14, 15, 19, 20, 25, 27, and 42. The details of pathogenic species with variant residues at specific positions are mentioned in the supplementary document Table S4. The MSA of the pathogenic sequences of the *CAP59* gene is illustrated in Figure 1b.

The MSA of non-pathogenic *CAP59* gene sequences indicated overall conservation, with specific variable residues at different positions (Figure 1c). The MSA of both pathogenic and non-pathogenic sequences showed alignment from positions 102 to 295, with conserved and semi-conserved residues mentioned in Figure 1d.

Based on these findings, pathogenic *CAP59* genes interact with a smaller, highly conserved region of the ancestral gene. Most pathogenic sequences are conserved, with specific variable residues at positions 10, 11, 14, 15, 19, 20, 25, 27, and 42, indicating key sites for functional divergence between species. Residues at these positions differ between species, indicating species-specific differences in the *CAP59* gene that may influence pathogenicity. This variability at specific positions may have functional consequences, affecting protein structure or interactions. The MSA of non-pathogenic sequences prioritizes conservation, demonstrating a stable genetic makeup in non-pathogenic species.

The MSA of both pathogenic and non-pathogenic sequences revealed alignment between positions 102 and 295, indicating a common genetic background. Residues in this region were conserved or semi-conserved, indicating possible functional similarities. Differences between pathogenic and non-pathogenic sequences provided information about *CAP59* gene evolution and strain divergence. Variable residues may correspond to important functional domains involved in pathogenicity. Further research in these areas may improve understanding of specific functions.

### Phylogenetic Analysis

The results showed that pathogenic and non-pathogenic species shared a common ancestor, which caused their divergence. Pathogenic species, such as *C. gattii*-VGIV and *C. gattii*-VGI, had a common ancestor, while *C. neoformans* and *C. gatti*-VGIV/VGIIIc, and *C. gatti*-VGII and *C. gatti*-VGIIb had different ancestor. Notably, the ancestors of *C. gattii*-VGII and *C. gattii*-VGIIb were descended from the *C. gattii* ancestor.

Moreover, the non-pathogenic species, *C. floricola* and *C. wingfieldii*, were closely related and descended from a common ancestor, while the ancestor of *C. amylolentus* was a descendent of *C. depauperatus* ancestor. Lastly, the ancestors of *C. floricola* and *C. wingfieldii* were the descendants of the *C. amylolentus* ancestor, as shown in Figure 1e.

The pairwise distance matrix indicated closeness among species. In pathogenic species, several pairs (*C. gattii* and *C. gattii*-VGII, *C. gattii* and *C. gattii*-VGIIb, and *C. gattii*-VGII and *C. gattii*-VGIIb) had a minimum distance of zero, signifying their close relation. 

Among non-pathogenic species, *C. floricola* and *C. wingfieldii* were the closest (distance: 0.0018885747). In addition, *C. amylolentus* showed a minimum distance of 0.1704998178 with three pathogenic species (*C. gattii*-VGI, *C. gattii*, and *C. neoformans*). The pairwise distance matrix for all species is mentioned in supplementary document Table S5.

### Gene Structures Retrieval

MODELLER was used to model the retrieved sequences through homology modeling, with AlphaFold structures of *C. neoformans* and *C. wingfieldii* serving as templates for other species. *C. gattii*-VGIV had the best Discrete Optimized Protein Energy (DOPE) score among pathogenic species (-21346.68164), while *C. amylolentus* had the best DOPE score among non-pathogenic species (-59030.45703). Furthermore, the predicted gene structures of the pathogenic and non-pathogenic species are shown in Figure 1 (f-p), while their DOPE scores are mentioned in supplementary document Table S6.

### Visualization and Superimposition of the Structures

Pathogenic species structures were superimposed using PyMOL, with conserved regions in grey and mutant regions in red (Figure 1q). Non-pathogenic species aligned regions with the pathogenic sequence in grey and non-aligned regions in pink (Figure 1r). Pathogenic species show mutations in the most conserved regions, leading to unique implications for the protein structure, function, and pathogenicity of each species.

## Discussion

*Cryptococcus* species can cause illnesses in both immunocompromised and immunocompetent people. *C. neoformans* and *C. gattii* are the primary species responsible for cryptococcosis in animals and humans, ranging from asymptomatic to severe and fatal meningitis [ 35 ]. However, the non-pathogenic *Cryptococcus* species are closely related to the pathogenic species [ 36 ]. Hence, understanding the molecular mechanisms that separate the pathogenic and non-pathogenic *Cryptococcus* species is essential for ascertaining the divergence and potential implications for the virulence of various species [ 37 ].

A protein family analysis revealed that pathogenic species (*C. neoformans*, *C. gattii*-VGI, and *C. gattii*-VGIV/VGIIIc) have an additional ligand binding region, which is absent in non-pathogenic species. Motif analysis revealed conserved regions in pathogenic species at the same positions, indicating high sequence similarity, while non-pathogenic species had motifs at different positions and longer sequence lengths.

The MSA analysis of *CAP59* gene sequences revealed that pathogenic strains primarily use a small, conserved region of the ancestral gene essential for function. Specific variable residues at positions 10, 11, 14, 15, 19, 20, 25, 27, and 42 indicate potential functional adaptation sites across species that lead to observed pathogenicity differences. Species-specific variations in these residues highlight differences in the *CAP59* gene.

The identified variability may influence protein structure and interactions, with functional implications observed in structural comparisons of pathogenic and non-pathogenic species. Non-pathogenic sequences exhibit overall conservation, indicating genetic stability. Alignment of both sequences reveals a shared genetic background in a specific region (positions 102–295), with conserved or semi-conserved residues, implying potential functional similarities. The differences provided insight into the evolutionary history of *CAP59*, with variable residues potentially corresponding to important functional domains. Further research in these areas could improve understanding of specific gene functions related to pathogenicity.

The phylogenetic analysis revealed close relationships in pairwise distance matrices between pathogenic species of *C. gattii* and *C. gattii*-VGII, *C. gattii* and *C. gattii*-VGIIb, and *C. gattii*-VGII and *C. gattii*-VGIIb. This closeness, supported by the phylogenetic tree, points to a common ancestor in their evolution. Similarly, among non-pathogenic species, *C. floricola* and *C. wingfieldii* were closely related in both the pairwise distance matrix and the phylogenetic tree, suggesting a common ancestor.

Moreover, the pairwise distance matrix revealed a close relationship between *C. amylolentus* (non-pathogenic) and three pathogenic species (*C. gattii*-VGI, *C. gattii*, and *C. neoformans*). This supports a study that suggested pathogenic species evolved from non-pathogenic *C. amylolentus* species, highlighting their close relationship in the pairwise distance matrix.

Moreover, the superimpositions of the pathogenic and non-pathogenic species were performed separately for both species, indicating a change in the structural conformation of the pathogenic species from 10 to 27 amino acid positions, which was the same region where the conserved, semi-conserved, and non-conserved residues were found, showing the difference in the level of the pathogenicity of the species.

Subsequently, this suggests that non-pathogenic species have longer *CAP59* gene sequences than pathogenic ones, possibly contributing to their non-virulence despite being closely related. Pathogenic sequences display major identity, with a small conserved region containing potential reasons for virulence and shorter sequence lengths, compared to non-pathogenic species.

## Conclusion

This study investigated *CAP59* gene evolution in *Cryptococcus*, which is crucial for virulence. Computational analyses revealed key differences, namely unique ligand binding in pathogenic species, conserved motifs, and variable residues influencing pathogenicity. Phylogenetic analysis suggested shared ancestry between pathogenic and non-pathogenic species, supporting the idea of pathogenic strains evolving from non-pathogenic ancestors, as seen in *C. amylolentus*. Structural comparisons showed significant conformational differences, especially in regions with conserved residues. Specific variable residues suggested potential sites for functional adaptation across species, contributing to observed pathogenicity differences. Shorter *CAP59* gene lengths and identical residues in pathogenic species may enhance virulence. This analysis deepened our understanding of *Cryptococcus* virulence factors, providing potential targets for further research.

## References

[1] Maziarz EK, Perfect JR ( 2016). Cryptococcosis. Infect Dis Clin North Am.

[2] Khawcharoenporn T, Apisarnthanarak A, Mundy LM ( 2007). Non-neoformans Cryptococcal Infections: a Systematic Review. Infection.

[3] Araujo G de S, Fonseca FL, Pontes B, Torres A, Cordero RJB, Zancopé-Oliveira RM, et al ( 2012). Capsules from Pathogenic and Non-Pathogenic Cryptococcus spp. Manifest Significant Differences in Structure and Ability to Protect against Phagocytic Cells. PLOS ONE.

[4] Gnat S, Łagowski D, Nowakiewicz A, Dyląg M ( 2021). A global view on fungal infections in humans and animals: opportunistic infections and microsporidiosis. J Appl Microbiol.

[5] Ghanem H, Sivasubramanian G ( 2021). Cryptococcus neoformans Meningoencephalitis in an Immunocompetent Patient after COVID-19 Infection. Case Rep Infect Dis.

[6] Farrer RA, Chang M, Davis MJ, van Dorp L, Yang DH, Shea T, et al ( 2019). A New Lineage of Cryptococcus gattii (VGV) Discovered in the Central Zambezian Miombo Woodlands. mBio.

[7] Gushiken AC, Saharia KK, Baddley JW ( 2021). Cryptococcosis. Infect Dis Clin.

[8] Montoya MC, Magwene PM, Perfect JR ( 2021). Associations between Cryptococcus Genotypes, Phenotypes, and Clinical Parameters of Human Disease: A Review. J Fungi.

[9] Zavala S, Baddley JW ( 2020). Cryptococcosis. Semin Respir Crit Care Med.

[10] Mada PK, Jamil RT, Alam MU ( 2023). Cryptococcus. In: StatPearls [Internet]..

[11] Akaihe CL, Nweze EI ( 2021). Epidemiology of Cryptococcus and Cryptococcosis in Western Africa. Mycoses.

[12] Diniz-Lima I, Fonseca LM da, Silva-Junior EB da, Guimarães-de-Oliveira JC, Freire-de-Lima L, Nascimento DO, et al ( 2022). Cryptococcus: History, Epidemiology and Immune Evasion. Appl Sci.

[13] Rathore SS, Sathiyamoorthy J, Lalitha C, Ramakrishnan J ( 2022). A holistic review on Cryptococcus neoformans. Microb Pathog.

[14] Casadevall A, Coelho C, Cordero RJB, Dragotakes Q, Jung E, Vij R, et al ( 2019). The capsule of Cryptococcus neoformans. Virulence.

[15] Zhao Y, Lin J, Fan Y, Lin X ( 2019). Life Cycle of Cryptococcus neoformans. Annu Rev Microbiol.

[16] Yang DH, England MR, Salvator H, Anjum S, Park YD, Marr KA, et al ( 2021). Cryptococcus gattii Species Complex as an Opportunistic Pathogen: Underlying Medical Conditions Associated with the Infection. mBio.

[17] Coelho C, Farrer RA ( 2020). Pathogen and host genetics underpinning cryptococcal disease. Adv Genet.

[18] Guterres DC, Ndacnou MK, Saavedra-Tobar LM, Salcedo-Sarmiento S, Colmán AA, Evans HC, et al ( 2021). Cryptococcus depauperatus, a close relative of the human pathogen C. neoformans, is associated with coffee leaf rust (Hemileia vastatrix) in Cameroon. Braz J Microbiol.

[19] Passer AR, Clancey SA, Shea T, David-Palma M, Averette AF, Boekhout T, et al ( 2022). Obligate sexual reproduction of a homothallic fungus closely related to the Cryptococcus pathogenic species complex. eLife [Internet]..

[20] Findley K, Rodriguez-Carres M, Metin B, Kroiss J, Fonseca Á, Vilgalys R, et al ( 2009). Phylogeny and Phenotypic Characterization of Pathogenic Cryptococcus Species and Closely Related Saprobic Taxa in the Tremellales. Eukaryot Cell.

[21] Sun S, Yadav V, Billmyre RB, Cuomo CA, Nowrousian M, Wang L, et al ( 2017). Fungal genome and mating system transitions facilitated by chromosomal translocations involving intercentromeric recombination. PLoS Biol.

[22] Passer AR, Coelho MA, Billmyre RB, Nowrousian M, Mittelbach M, Yurkov AM, et al ( 2019). Genetic and Genomic Analyses Reveal Boundaries between Species Closely Related to Cryptococcus Pathogens. mBio.

[23] Findley K, Sun S, Fraser JA, Hsueh YP, Averette AF, Li W, et al ( 2012). Discovery of a Modified Tetrapolar Sexual Cycle in Cryptococcus amylolentus and the Evolution of MAT in the Cryptococcus Species Complex. PLoS Genet.

[24] Farrer RA, Desjardins CA, Sakthikumar S, Gujja S, Saif S, Zeng Q, et al ( 2015). Genome Evolution and Innovation across the Four Major Lineages of Cryptococcus gattii. mBio.

[25] Huang Y, Zang X, Yang C, Deng H, Ma X, Xie M, et al ( 2022). Gene, virulence and related regulatory mechanisms in Cryptococcus gattii. Acta Biochim Biophys Sin.

[26] The UniProt Consortium ( 2023). UniProt: the Universal Protein Knowledgebase in 2023. Nucleic Acids Res.

[27] Wheeler D, Bhagwat M ( 2007). BLAST QuickStart. In: Comparative Genomics: Volumes 1 and 2 [Internet]..

[28] Mistry J, Chuguransky S, Williams L, Qureshi M, Salazar GA, Sonnhammer ELL, et al ( 2021). Pfam: The protein families’ database in 2021. Nucleic Acids Res.

[29] Paysan-Lafosse T, Blum M, Chuguransky S, Grego T, Pinto BL, Salazar GA, et al ( 2023). InterPro in 2022. Nucleic Acids Res.

[30] Bailey TL, Williams N, Misleh C, Li WW ( 2006). MEME: discovering and analyzing DNA and protein sequence motifs. Nucleic Acids Res.

[31] Sievers F, Wilm A, Dineen D, Gibson TJ, Karplus K, Li W, et al ( 2011). Fast, scalable generation of high-quality protein multiple sequence alignments using Clustal Omega. Mol Syst Biol.

[32] Tamura K, Stecher G, Kumar S ( 2021 ). MEGA11: Molecular Evolutionary Genetics Analysis Version 11. Mol Biol Evol.

[33] ( 2003). Highly accurate protein structure prediction with AlphaFold | Nature [Internet].

[34] Yuan S, Chan HCS, Hu Z ( 2017). Using PyMOL as a platform for computational drug design. WIREs Comput Mol Sci.

[35] Campbell LT, Simonin AR, Chen C, Ferdous J, Padula MP, Harry E, et al ( 2015). Cryptococcus Strains with Different Pathogenic Potentials Have Diverse Protein Secretomes. Eukaryot Cell.

[36] Gerstein AC, Nielsen K ( 2017). It’s not all about us: evolution and maintenance of Cryptococcus virulence requires selection outside the human host. Yeast.

[37] Gupta S, Paul K, Kaur S ( 2020). Diverse species in the genus Cryptococcus: Pathogens and their non-pathogenic ancestors. IUBMB Life.

